# A fungal phylogeny based on 42 complete genomes derived from supertree and combined gene analysis

**DOI:** 10.1186/1471-2148-6-99

**Published:** 2006-11-22

**Authors:** David A Fitzpatrick, Mary E Logue, Jason E Stajich, Geraldine Butler

**Affiliations:** 1 School of Biomolecular and Biomedical Science, Conway Institute, University College Dublin, Belfield, Dublin 4, Ireland; 2 Department of Molecular Genetics and Microbiology, Duke University, Durham, North Carolina 27708, USA

## Abstract

**Background:**

To date, most fungal phylogenies have been derived from single gene comparisons, or from concatenated alignments of a small number of genes. The increase in fungal genome sequencing presents an opportunity to reconstruct evolutionary events using entire genomes. As a tool for future comparative, phylogenomic and phylogenetic studies, we used both supertrees and concatenated alignments to infer relationships between 42 species of fungi for which complete genome sequences are available.

**Results:**

A dataset of 345,829 genes was extracted from 42 publicly available fungal genomes. Supertree methods were employed to derive phylogenies from 4,805 single gene families. We found that the average consensus supertree method may suffer from long-branch attraction artifacts, while matrix representation with parsimony (MRP) appears to be immune from these. A genome phylogeny was also reconstructed from a concatenated alignment of 153 universally distributed orthologs. Our MRP supertree and concatenated phylogeny are highly congruent. Within the Ascomycota, the sub-phyla Pezizomycotina and Saccharomycotina were resolved. Both phylogenies infer that the Leotiomycetes are the closest sister group to the Sordariomycetes. There is some ambiguity regarding the placement of *Stagonospora nodurum*, the sole member of the class Dothideomycetes present in the dataset.

Within the Saccharomycotina, a monophyletic clade containing organisms that translate CTG as serine instead of leucine is evident. There is also strong support for two groups within the CTG clade, one containing the fully sexual species *Candida lusitaniae*, *Candida guilliermondii *and *Debaryomyces hansenii*, and the second group containing *Candida albicans*, *Candida dubliniensis*, *Candida tropicalis*, *Candida parapsilosis *and *Lodderomyces elongisporus*. The second major clade within the Saccharomycotina contains species whose genomes have undergone a whole genome duplication (WGD), and their close relatives. We could not confidently resolve whether *Candida glabrata *or *Saccharomyces castellii *lies at the base of the WGD clade.

**Conclusion:**

We have constructed robust phylogenies for fungi based on whole genome analysis. Overall, our phylogenies provide strong support for the classification of phyla, sub-phyla, classes and orders. We have resolved the relationship of the classes Leotiomyctes and Sordariomycetes, and have identified two classes within the CTG clade of the Saccharomycotina that may correlate with sexual status.

## Background

Traditional methods of systematics based on morphology of vegetative cells, sexual states, physiological responses to fermentation and growth tests can assign fungal species to particular genera and families. However, higher-level relationships amongst these groups are less certain and are best elucidated using molecular techniques. Today single-gene phylogenies (especially 18S ribosomal DNA-based ones) have established many of the accepted relationships between fungal organisms. The benefits of the 18S rDNA approach are the vertical transmission of this gene, its ubiquity and the fact that it has slowly evolving sites. However, single-gene analyses are dependent on the gene having an evolutionary history that reflects that of the entire organism, an assumption that is not always true. It has been estimated that there are approximately 1.42 million fungi species yet to be discovered [1,2]. It follows that it is essential that we develop methods to infer a robust phylogeny of known taxonomic groups, so we can provide a framework for future studies.

Between 1990 and 2003, 560 fungal research papers reporting phylogenies were published, of which about 84% were derived using rDNA [3]. Protein coding genes are rarely used in fungal phylogenetics but when used they have the ability to resolve deep level phylogenetic relationships [4]. Phylogeny reconstruction based on a single gene may not be robust, as vital physiological processes and basic adaptive strategies do not always correlate with ribosomal derived trees [5]. Individual genes also contain a limited number of nucleotide sites and therefore limited resolution. An alternative approach to a single gene phylogeny is to combine all available phylogenetic data. There are two commonly used methods to do this: multigene concatenation and supertree analysis.

Multigene concatenation proposes that phylogenetic analysis should always be performed using all available character data, essentially sticking many aligned genes together to give a large alignment. Combining the data increases its informativeness, helps resolve nodes, basal branching and improve phylogenetic accuracy [6]. Gene concatenation has been justified on philosophical grounds, as it attempts to maximise the informativeness and explanatory power of the character data used in the analysis [7]. Numerous genome phylogenies have been derived by concatenation of universally distributed genes [8-13]. One advantage of concatenated phylogenies is that observed branch lengths are comparable across the entire tree, as they are derived from common proteins. This allows an objective, quantitative analysis of the consistency of traditional groupings [8]. However, gene concatenation also has some well-documented problems. For example, erroneous phylogenetic inferences can be made if recombination has occurred within the individual datasets used. Phylogenetic inference from sequence data can also be misled by systematic errors (e.g. compositional biases) [14]. These errors can be exacerbated when longer sequences are used, leading to strong support for inferences that in reality may be false.

A supertree analysis on the other hand generates a phylogeny from a set of input trees that possess fully or partially overlapping sets of taxa. Because the input trees need only overlap minimally, each source tree must share at least two taxa with one other source tree; more generally, supertree methods take as input a set of phylogenetic trees and return one or more phylogenetic trees that represent the input trees [15]. Because of the way supertrees summarise taxonomic congruence, they limit the impact of individual genes on the global topology and account for extensive differences in evolutionary rates and substitution patterns among genes in a gene-by-gene manner [16]. Therefore, we can get a phylogeny that is truly representative of the entire genome. Supertree techniques are slowly becoming commonplace in biology [17-22] and will play an important role in ascertaining the tree of life.

This study undertook a phylogenomic approach [23,24] to fungal taxonomy. Using both supertree and concatenated methods, all available fungal genomic data was analysed in an effort to address some long-standing questions regarding ancestry and sister group relationships amongst diverse fungal species.

## Results and discussion

### Genome data infers a robust fungal phylogeny

**Figure 1:**
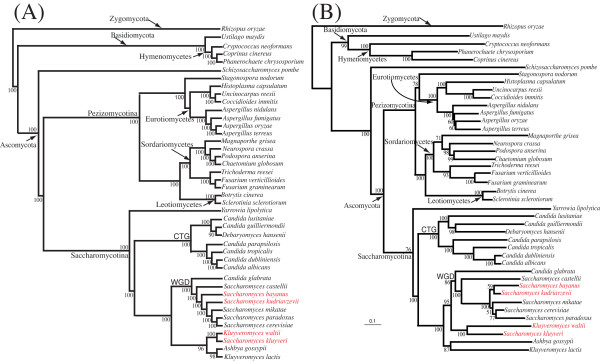
**MRP (A) and AV (B) fungal supertrees derived from 4,805 fungal gene families**. Bootstrap scores for all nodes are displayed. The AV supertree method makes use of input tree branch lengths. *Rhizopus oryzae *has been selected as an outgroup. The Basidiomycota and Ascomycota phyla form distinct clades. Subphyla and class clades are highlighted. Two clades of special interest include the node that contains the organisms that translate CTG as serine instead of leucine, and the node that contains the genomes that have undergone a genome duplication (WGD). Topological differences between supertree phylogenies are highlighted in red font.

Our dataset consisted of 345,829 protein-coding genes from 42 fungal genomes (Table 1). Overall we identified 4,805 putative orthologous gene families (see methods). Maximum likelihood (ML) phylogenies were reconstructed for individual gene families. These 4,805 trees were used as input data for our supertree analysis, constructed using three different methods: matrix representation with parsimony (MRP) [25,26], the average consensus method (AV) [27], and the most similar supertree analysis (MSSA) method [21]. All three methods inferred congruent phylogenies, all supertree results discussed here are based on the MRP and AV phylogenies (Figure 1A&B). The results for the MSSA supertrees can be found in additional material [see additional file 1]. The YAPTP (yet another permutation tail probability randomization) test [21], which tests the null hypothesis that congruence between the input trees is no better than random, was used to assess the degree of congruence between input trees. The distribution of the scores of the 100 optimal supertrees from the YAPTP test is between 84,184 – 84,464, whereas the original unpermuted data received a score of 27,686. These scores suggested that congruence across the input trees is greater than expected by chance (P > 0.01) [21,22] and we deemed the data suitable for supertree analyses.

**Table 1:** Fungal organisms used in this analysis are listed. Phylum, sub-phylum and classes are shown. *Gene sets were generated in house.

Species	Phlum	Genes	Citation or sequencing group
***Candida albicans ***	Ascomycota	6,662	[86]
Candida dubliniensis*	Ascomycota	6,027	Sanger Centre
Candida tropicalis*	Ascomycota	6,530	Broad-FGI
Candida parapsilosis*	Ascomycota	4,891	Sanger Centre
Candida lusitaniae*	Ascomycota	5,941	Broad-FGI
Candida guilliermondii*	Ascomycota	5,235	Broad-FGI
***Debaryomyces hansenii ***	Ascomycota	6,896	[49]
***Saccharomyces bayanus ***	Ascomycota	4,492	[87, 88]
***Saccharomyces castellii ***	Ascomycota	4,677	[87]
***Saccharomyces cerevisiae ***	Ascomycota	5,873	[89]
***Saccharomyces kudriavzevii ***	Ascomycota	3,768	[87]
***Saccharomyces mikatae ***	Ascomycota	4,525	[87, 88]
***Saccharomyces paradoxus ***	Ascomycota	4,788	[88]
***Candida glabrata ***	Ascomycota	5,272	[49]
***Kluyveromyces lactis ***	Ascomycota	5,331	[49]
***Saccharomyces kluyveri ***	Ascomycota	2,968	[87]
***Kluyveromyces waltii ***	Ascomycota	5,214	[90]
***Ashbya gossypii ***	Ascomycota	4,718	[91]
***Yarrowia lipolytica ***	Ascomycota	6,666	[49]
***Magnaporthe grisea ***	Ascomycota	11,109	[92]
***Neurospora crassa ***	Ascomycota	10,620	[93]
Podospora anserina *	Ascomycota	10,443	Broad-FGI
***Chaetomium globosum ***	Ascomycota	11,124	Broad-FGI
Trichoderma reesei *	Ascomycota	13,248	JGI-DOE
***Fusarium graminearum ***	Ascomycota	11,640	Broad-FGI
Fusarium verticillioides *	Ascomycota	12,751	Broad-FGI
***Aspergillus oryzae ***	Ascomycota	12,062	NITE (Japan)
***Aspergillus nidulans ***	Ascomycota	9,541	Broad-FGI
***Aspergillus fumigatus ***	Ascomycota	9,923	TIGR and Sanger Centre
Aspergillus terreus *	Ascomycota	10,285	Microbia
Uncinocarpus reesii *	Ascomycota	6,573	Broad-FGI
Histoplasma capsulatum *	Ascomycota	6,605	Broad-FGI
Coccidioides immitis *	Ascomycota	6,622	Broad-FGI
***Sclerotinia sclerotiorum ***	Ascomycota	14,522	Broad-FGI
***Botrytis cinerea ***	Ascomycota	16,448	Broad-FGI
***Stagonospora nodorum ***	Ascomycota	16,597	Broad-FGI
***Schizosaccharomyces pombe ***	Ascomycota	4,991	[94]

Coprinus cinereus*	Basidiomycota	9,452	Broad-FGI
Phanerochaete chrysosporium*	Basidiomycota	10,216	[95]
***Cryptococcus neoformans ***	Basidiomycota	6,594	[96]
***Ustilago maydis ***	Basidiomycota	6,522	Broad-FGI

***Rhizopus oryzae ***	Zygomycota	17,467	Broad-FGI

^a^Broad-FGI, MIT/Harvard Broad Institute, funded through the Fungal Genome Initiative; JGI-DOE, Department of Energy Joint Genome Institute, Walnut Creek, California; Microbia, Cambridge, Massachusetts; Sanger Caenter, Wellcome Trust Sanger Center, Hinxton, Cambridge, United Kingdom.

Presently there is a heated philosophical debate as to what is the best approach for reconstructing genome phylogenies. Instead of using supertree methods, some prefer to concatenate universally distributed genes. In an attempt to circumvent this argument we decided to use a global congruence [28] approach, where both ideologies are used and the resulting phylogenies are cross-corroborated.

**Figure 2:**
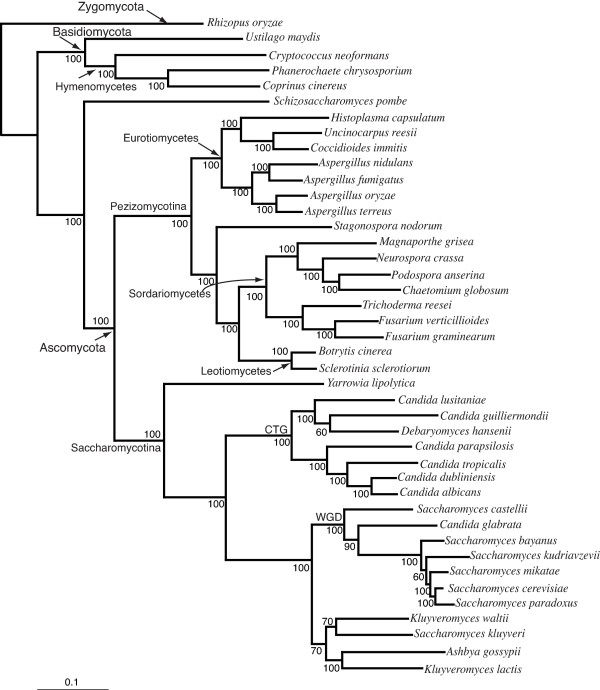
**Maximum likelihood phylogeny reconstructed using a concatenated alignment of 153 universally distributed fungal genes**. The concatenated alignment contains 42 taxa and exactly 38,000 amino acid positions. The optimum model according to ModelGenerator [85] was found to be WAG+I+G. The number of rate categories was 4 (alpha = 0.83) and the proportion of invariable sites was approximated at 0.20. Bootstrap scores for all nodes are displayed. *S. castellii *is found at the base of the WGD node.

From our analysis, we initially located 227 protein families that were universally distributed between all taxa. Seven of the genomes present in this analysis have undergone a genome duplication. In an effort to minimize the effects of hidden paralogy, we only considered genes that were found in conserved syntenic blocks for selected organisms (see methods). Overall 153 of the 227 gene families met these criteria, and were used for further analysis [see additional file 2]. These gene families were individually aligned and concatenated together to give an alignment of exactly 38,000 amino acids in length. A ML phylogeny was reconstructed (Figure 2) and compared to the supertree derived from 4,805 gene families (Figure 1). In the following discussion we use the phylum, sub-phylum and class taxonomic scheme of the NCBI taxonomy browser [29].

Overall, there is a high degree of congruence between supertree and concatenated alignment phylogenies (Figures 1 &2). Unsurprisingly all phylogenies inferred 3 strongly supported phyla branches, the Zygomycota, the Basidiomycota and the Ascomycota (Figures 1 &2).

The Basidiomycota form a well-supported clade. The three members of the Hymenomycetes class form a robust sub-group with 100% bootstrap support (BP). Within the Hymenomycetes there is a clade containing the two members {*Coprinus cinereus *and *Phanerochaete chrysosporium*} of the order Agaricales, separate from *Cryptococcus neoformans*, which belongs to the order Tremellales.

The majority of the species studied in this analysis belong to the Ascomycota phylum. Within the Ascomycota there are two main subphyla, the Pezizomycotina and Saccharomycotina. Both these groups form separate well-supported sub-phyla clades (Figures 1 &2). *Schizosaccharomyces pombe*, the only member of the Schizosaccharomycetes, sits outside these two sub-phyla clades.

Within the Pezizomycotina a number of well-defined class-clades are observed, namely the Sordariomycetes, the Leotiomycetes and Eurotiomycetes (Figures 1 &2). The relationship between these classes has been the subject of debate. Our supertrees and concatenated phylogenies infer that the Leotiomycetes and Sordariomycetes are sister classes. This agrees with the poorly supported rDNA based analysis of Lumbsch *et al *[30] but is in disagreement with Lutzoni *et al *[3], who based on a four gene combined dataset placed the Dothideomycetes as a sister group to the Sordariomycetes. The grouping of Leotiomycetes and Sordariomycetes in both our phylogenies is highly supported (100% BP) and is likely to represent the true relationship. Furthermore, a recent phylogenomic study of 17 Ascomycota genomes by Robbertse *et al *[12] reported similar inferences.

There is conflict however between our supertrees and concatenated phylogenies regarding the positioning of *Stagonospora nodorum *(the only representative of the Dothideomycetes lineage). The supertrees (Figure 1) place *S. nodorum *beside the Eurotiomycetes (100% BP), and supports the analysis of Lutzoni *et al *[3] who also group the Dothideomycetes and Eurotiomycetes lineages together. Conversely, our concatenated alignment (Figure 2) infers that *S. nodorum *is more closely related to the Sordariomycetes and Leotiomycetes lineages (100% BP). Based on their concatenated alignment Robbertse *et al *[12] have also reported conflicting inferences regarding the phylogenetic position of *S. nodorum *[12]. Their phylogenies reconstructed using neighbor joining and maximum likelihood methods inferred a sister group relationship between *S. nodorum *and Eurotiomycetes in line with our supertree inference. However a phylogeny inferred using maximum parsimony placed *S. nodorum *at the base of the Pezizomycotina [12]. To confidently resolve this incongruence additional Dothideomycetes genomes will be required.

Within the Eurotiomycetes class there is a clade corresponding to the order Onygenales {*Histoplasma capsulatum, Coccidioides immitis *and *Uncinocarpus reesii*}. The Onygenales clade is of interest as it contains *Coccidioides immitis*. This organism was initially classified as a protist [31] but further research showed it was fungal, and separate studies placed it in three different divisions of Eumycota [32-34]. Subsequent ribosomal phylogeny studies [35,36] suggested a close phylogenetic relationship between *C. immitis *and *U. reesii *to the exclusion of *H. capsulatum*. Our supertrees and concatenated phylogenies based on whole genome data concur with the placement of *C. immitis *and *U. reesii *as sister taxa.

The Eurotiomycetes branch containing the *Aspergillus *clade is also of interest, as supertree and concatenated phylogenies infer that *A. oryzae *and *A. terreus *are each others closest relatives (Figures 1 &2) (100% BP respectively). A minor difference between the supertrees and concatenated phylogenies regards the phylogenetic position of *A. nidulans *and *A. fumigatus*. The concatenated alignment infers that these organisms are sister taxa (100% BP), the supertrees fails to make this inference and instead positions *A. fumigatus *beside the {*A. oryzae*, *A. terreus*} clade with 100% BP.

A number of subclass clades are evident in the Sordariomycetes clade. For example *Fusarium graminearum*, *Fusarium verticilliodes *and *Trichoderma reesei *belong to the subclass Hypocreomycetidae. Similarily *Neurospora crassa*, *Chaetomium globosum *and *Podospora anserina *all belong to the subclass Sordariomycetidae. The inferred phylogenetic relationships amongst the Sordariomycetidae organisms concurs with previous phylogenetic studies [37].

### Relationships within the Saccharomycotina lineage

Overall the MRP and AV supertree topologies (Figure 1A&B) are very similar. A noticeable difference occurs in the branch directly adjacent to the WGD clade. The MRP tree (and the concatenated phylogeny (Figure 2)) places the grouping of {*K. waltii*, *S. kluyveri*} and {*K. lactis*, *A. gossypii*} as sister branches, while the AV supertree infers that {*K. waltii*, *S. kluyveri*} are closer to the WGD clade than to the {*K. lactis*, *A. gossypii*} clade. Recently Jeffroy *et al *[38] constructed a multigene phylogeny (using 13 of the 42 species included in our analysis) that is congruent with our MRP supertree for these species. They state that *K. lactis *and *A. gossypii *are evolving faster than *S. kluyveri *and *K. waltii *and are therefore likely to be "attracted" to long branches. The AV method makes use of branch length information from individual gene trees, and we suspect the inferred AV supertree phylogeny amongst the {*K. lactis*, *A. gossypii*} and {*S. kluyveri*, *K. waltii*} clades may be suffering from long-branch attraction artifacts. As additional taxa can help break long branches, it is likely that stochastic errors will be eradicated with the addition of extra genome data when it becomes available, thus eliminating erroneous inferences.

The sister group relationships amongst the *Saccharomyces sensu stricto *species also differs between our supertree phylogenies (Figure 1A&B). For example, the MRP phylogeny places *S. bayanus *at the base of the *Saccharomyces sensu stricto *node and infers a ladderised topology amongst the *Saccharomyces sensu stricto *species. The MRP inferences (Figure 1A) match those proposed by our multigene phylogeny (Figure 2) and are identical to that proposed by Jeffroy *et al*. Alternatively, the AV supertree infers that *S. bayanus *and *S. kudriavzevii *are sister taxa (Figure 1B). There is also an interesting difference regarding the relative position of *Candida glabrata *and *Saccharomyces castellii*, the supertrees and the multigene phylogeny constructed by Jeffroy *et al *[38] place *C. glabrata *at the base of the clade containing the organisms that have undergone a WGD (Figure 1A). Alternatively, our concatenated alignment infers a phylogeny with *S. castellii *at the base of the WGD clade (Figure 2), in agreement with syntenic studies [39].

**Figure 3:**
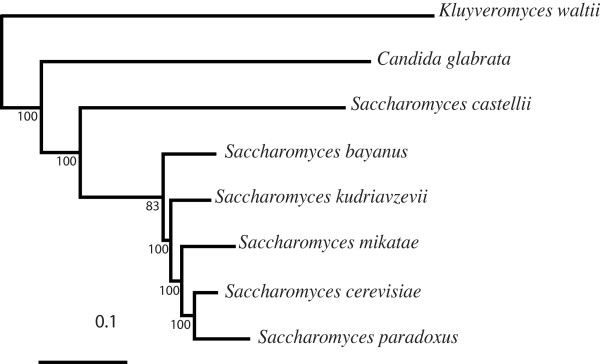
Average consensus supertree of WGD-specific clade inferred from 1,368 underlying phylogenies. MRP and MSSA supertrees are identical. Bootstrap scores are shown at all nodes. Bayesian analysis of recoded protein alignments and further supertree analysis yielded identical results.

It is possible that the differences between the phylogenies inferred by the MRP and AV supertrees for the *Saccharomyces sensu stricto *group are due the inclusion of paralagous sequences from the WGD species. We therefore constructed a supertree based exclusively on the species that have undergone the WGD, using 1,368 putative orthologous gene families (see methods). ML phylogenies were reconstructed for all gene families. The WGD-specific supertree (Figure 3) concurs with the MRP fungal supertree (Figure 1A) and the phylogeny of Jeffroy *et al*, suggesting this topology is correct.

The placement of *C. glabrata *as the most basal WGD genome is in disagreement with the tree inferred from the concatenated alignment (Figure 2). We therefore investigated the influence of fast evolving sites. Using a gamma distribution, we placed fast-evolving sites for each gene family into one of 8 categories, where site class 8 was the most heterogeneous, and class 1 were stationary. We systematically removed the fastest evolving sites one at a time, and rebuilt ML phylogenies based on these reduced alignments. Supertrees were once again reconstructed for these new phylogeny sets. When the two fastest classes of sites were removed, (reducing the combined length of all 1,368 genes by ~18% and ~30%), the resultant supertrees group *S. castelli *and *C. glabrata *as a monophyletic group and fail to differentiate which is closer to the outgroup [see additional file 3]. When we additionally remove the third fastest evolving site class (reducing the combined length by ~38%), the final supertree [see additional file 3] again infers *C. glabrata *at the base of the WGD clade (Figure 3). In an effort to account for compositional biases we also recoded the underlying amino acid alignments into the six Dayhoff groups and inferred individual gene phylogenies using the Bayesian criterion [see additional file 4]. The resultant supertree is identical to that shown in Figure 3, and again places *C. glabrata *at the base of the WGD clade.

**Figure 4:**
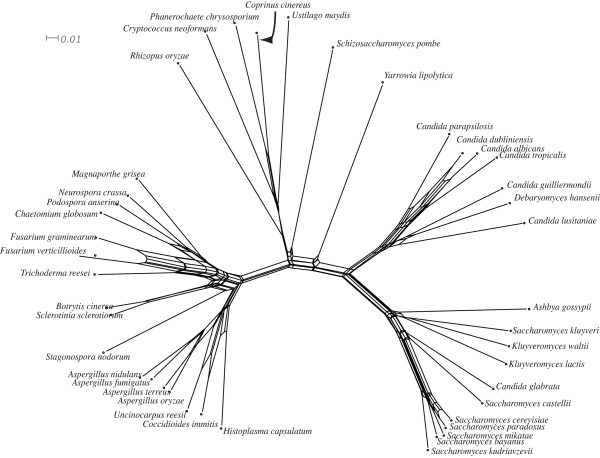
**Phylogenetic network reconstructed using a concatenated alignment of 153 universally distributed fungal genes**. The NeighborNet method was used to infer splits within the alignment. For display purposes bootstrap scores are not shown [see additional file 5].

To analyse the degree of conflicting phylogenetic signal within the concatenated alignment, a phylogenetic network was constructed (Figure 4). Numerous alternative splits are present (491 in total). A bootstrap analysis was preformed on the phylogenetic network [see additional file 5]. It is interesting to note that we never observe a split that excludes either *C. glabrata *or *S. castellii *from the remaining WGD organisms. This conflicts with the concatenated phylogeny (Figure 2), which strongly infers that *C. glabrata *sits beside the remaining WGD organisms to the exclusion of *S. castellii*. It is possible that a systematic bias [40] may be influencing our supertrees, as synteny information clearly shows that *S. castellii *diverges from the *Saccharomyces sensu stricto *lineage before *S. castellii*, [39]. Therefore topologies that place *C. glabrata *as an outgroup to the *Saccharomyces sensu stricto *lineage and *S. castellii *are unreliable [39] and need closer scrutiny. These incongruences suggest that genome data for additional basal WGD species is required to confidently resolve inferences at the base of the WGD clade.

### Phylogenetic relationships amongst *Candida *species

Both super tree (Figure 1) and superalignment (Figure 2) topologies inferred a robust monophyletic clade containing organisms which translate CTG as serine instead of leucine [41-44]. This codon reassignment has been proposed to have occurred ~170 million years ago [45]. Further inspection showed that there are two distinct CTG sub-clades, the first contains {*Candida lusitaniae*, *Candida guilliermondii*, *Debaromyces hansenii*} and the second containing {*Candida tropicalis*, *Candida albicans*, *Candida dubliniensis*, *Candida parapsilosis*} (Figure 1). *C. lusitaniae *and *C. guilliermondii *are haploid yeasts, and are apparently fully sexual [46-48]. *D. hansenii *is homothallic, with a fused mating locus [49,50]. In contrast, members of the second clade have at best a cryptic sexual cycle and have never been observed to undergo meiosis [51-55]. We decided to investigate this clade in further detail, and performed specific supertree, spectral and network analyses. Trace sequence data for *Lodderomyces elongisporus*, once proposed as the sexual form (teleomorph) of *C. parapsilosis *were also included [56,57].

**Figure 5:**
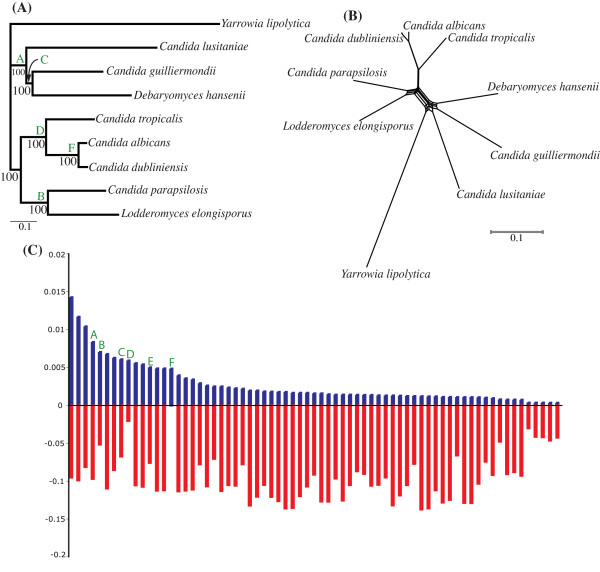
Average consensus supertree of CTG specific clade (A). *Y. lipolytica *was chosen as an outgroup. Bootstrap scores are shown at all nodes. (B) A phylogenetic network of 1,208 concatenated genes was inferred with the NeighborNet method. The topologies of CTG-clade specific supertree and network are congruent. (C) Spectral analysis of the concatenated alignment). Bars above the x-axis represent frequency of support for each split. Bars below the x-axis represent the sum of all corresponding conflicts. Letters above columns represent particular splits in the data, and where applicable these have also been mapped onto the supertree.

We located 2,146 putative orthologous gene families from our CTG database (see methods). ML phylogenies were reconstructed for all gene families, and a supertree based on these trees was reconstructed. The resultant CTG specific supertree placed *L. elongisporus *within the asexual clade (Figure 5A) with high BP support (100%), in agreement with other phylogenetic studies [58,59]. A CTG specific phylogenetic network was also constructed and infers that *L. elongisporus *groups beside *C. parapsilosis*, although there is a degree of conflict with this inference illustrated by a number of alternative splits (Figure 5B). Interestingly there is no conflict for the grouping of *C. albicans *and *C. dubliniensis *illustrating their high genotypic similarity [60]. These results raise interesting questions regarding the sexual status of the *Candida *species. It is possible that the "asexual" species are in fact fully sexual. *C. albicans *and *C. dubliniensis *have been observed to mate [53], and in addition the *C. albicans *genome contains most of the requirements for meiosis [61]. In contrast the evidence that *L. elongisporus *reproduces sexually is sketchy, and is based on the appearance of asci, with one (or sometimes two) spores [62]. It is clear that further analysis is required, which will be greatly aided when the fully annotated genome sequences of *L. elongisporus *and *C. parapsilosis *become available.

Our CTG specific supertree also suggests that *D. hansenii *and *C. guilliermondii *are sister taxa, as they are grouped together with high support (100% BP) to the exclusion of *C. lusitaniae*. Other studies [58,63] have placed *C. lusitaniae *in a clade beside *C. guilliermondii*, and inferred a closer relationship between the two compared with *Debaryomyces *species. We found 1,208 gene families present in all CTG taxa; these were concatenated together to give a nucleotide alignment of 1,291,068 sites or 860,712 sites when third codon positions are removed. A phylogenetic network based on this nucleotide alignment (Figure 5B) corroborated the CTG-specific supertree regarding the grouping of *C. guilliermondii *and *D. hansenii *as sister taxa to the exclusion of *C. lusitianiae*. Subsequent spectral analyses (Figure 5C) reinforce our CTG specific supertree and network inferences. For example, split A (Figure 5C) shows the relatively high degree of support for the grouping of three sexual species {*C. lusitianiae, C. guilliermondii and D. hansenii*} as sister taxa. Split C groups *C. guilliermondii *and *D. hansenii *together, in agreement with our CTG supertree and network. However, there is nearly equal character support for the grouping of *C. lusitaniae *and *D. hansenii *(0.00609 vs. 0.00501) illustrated by split E (Figure 5C). Therefore, based on whole genome comparisons there is only marginal evidence for the grouping of *C. guilliermondii *with *D. hansenii *to the exclusion of *C. lusitianiae*.

## Conclusion

In this study we set out to reconstruct a fungal phylogeny from whole genome sequences. Two alternative strategies were chosen (supertrees and concatenated methods), and overall we observed a high degree of congruence between both approaches. We recovered robust fungal, phyla, sub-phyla and class clades. Overall our inferences agreed with previous phylogenetic studies based on single genes and morphological characteristics.

The phylogenomic approach undertaken in this study is novel in fungal phylogenetics as it circumvents problems associated with single gene phylogenies and selection of robust phylogenetic markers. Our results suggest that it may be possible to piece together the tree of life using whole genomes. This is of interest as we expect the number of available genomes to increase substantially in tandem with new sequencing strategies [64], which continue to decrease the costs associated with sequencing. However, our study also shows that certain nodes of the tree (such as the WGD clade) are difficult to resolve even with genome scale data.

## Methods

### Sequence data

The fungal database used in this analysis consisted of 42 genomes (Table 1). Of these 28 are complete and gene datasets are available. Gene annotation for genomes with no annotations was performed using two separate approaches. The first involved a reciprocal best BLAST [65] search with a cutoff E-value of 10^-7 ^of *Candida albicans *protein coding genes against unannotated *Candida *genomes (Table 1). Top BLAST hits longer than 300 nucleotides were retained as putative open reading frames. The second approach involved a pipeline of analysis that combined several different gene prediction programs including *ab initio *programs SNAP [66], Genezilla [67], and AUGUSTUS [67] with gene models from Exonerate [68] and Genewise [69] based on alignments of proteins and Expressed sequence tags. The lines of evidence were merged into a single gene prediction using a combiner GLEAN (AJ Mackey, Q Liu, FCN Pereira, DS Roos, unpublished data). These annotations are freely available for download [70].

### Reconstruction of individual gene trees

Fungal homologous sequences were identified using the BLASTP algorithm [65] with a cutoff E-value of 10^-7 ^by randomly selecting a sequence from the database, finding its homologs, and removing the entire family from the database. Another randomly selected sequence from within the reduced database was then used as the new starting point for the next search. This procedure was repeated until all sequences had been removed from the database. Gene families with more than one representative from any species were not considered for further analysis. Those remaining families with a minimum of four sequences, and longer than 100 amino acids in length were selected for phylogenetic analysis. In total 5,316 protein families met these criteria. Individual protein families were aligned using ClustalW 1.81 [71] with the default settings. The average length of each protein alignment was 697 sites. Due to the large number of protein families it was not possible to manually curate all alignments. We therefore used only conserved alignment blocks, located using Gblocks version 0.91 b [72]. This filtering stage reduced the average length of our alignments to 214 sites. Permutation tail probability tests (PTP) [73,74] were performed on each alignment to test for the presence of evolutionary signal better than random (P < 0.01). We found that 511 alignments failed the PTP test; therefore 4,805 were used for phylogenetic reconstruction analysis. Using MultiPhyl [75] appropriate protein substitution models were selected and used to reconstruct ML phylogenies for each individual gene family. Bootstrap resampling was carried out 100 times on each alignment and the results were summarised with the majority-rule consensus method with a threshold of 70%. These phylogenies were used as input data in our supertree analysis. To account for possible compositional biases within our data, neighbor joining [76] phylogenies were also reconstructed based on distances derived from the LogDet transformation [77].

We were concerned that our strategy for locating orthologous gene families was too liberal. Therefore, we also utilised a second stricter database search strategy that located 809 gene families [see additional file 1 &additional file 6].

### Supertree reconstruction

In total 4,805 input trees were used as source data for this supertree analysis. Using the supertree software package CLANN 3.0.3b1 [78] three supertree methods were used to reconstruct fungal phylogenies, the average consensus method (AV) [27], the most similar supertree analysis (MSSA) method [21], and matrix representation with parsimony (MRP) [25,26]. Using CLANN 3.0.3b1, 100 bootstrap resamplings were also carried out on the input data. We tested for the presence of signal within our data using the YAPTP test.

### Multigene analysis

All proteins from the genome sequences were compared with FASTP [79] to find orthologous genes via a best bi-directional strategy. The ortholog sets for each pair of species were combined with single-linkage clustering to form multi-gene clusters of orthologs. In order to identify a set of single-copy genes across all organisms, only those clusters with exactly one member per species were considered for further analysis, we located 227 protein families that contain all fungal taxa. To help identify ohnologs and possible paralogs (with reference to the genomes that have undergone a genome duplication) we used the yeast genome browser [80,81] to filter out genes that have no syntenic evidence. Overall 153 gene families were used for further analysis [see additional file 2]. Individual gene families were aligned, manually edited and concatenated together to yield an alignment with 38,000 amino acid sites. A ML phylogeny was reconstructed for this alignment using the MultiPhyml software. Branch supports were determined via bootstrapping. In an attempt to visualise the degree of phylogenetic conflict within this concatenated alignment a phylogenetic network was generated using the NeighborNet method [82].

### Investigation of specific clades

#### CTG clade

The genomes of *C. albicans*, *C. dubliniensis*, *C. tropicalis*, *C. parapsilosis*, *D. hansenii*, *C. guilliermondii*, *C. lusitaniae *and the outgroup *Y. lipolytica *were combined to give a CTG specific database. Data for *L. elongisporus *was retrieved from the NCBI trace database and coding genes were predicted using a reciprocal best BLASTP search against *C. albicans*. In total 2,146 gene families were longer than 100 amino acids in length, with evolutionary signal, were retained for supertree analysis. ML phylogenies were reconstructed for all gene families as described above, and representative supertrees were reconstructed. A concatenated alignment based on 1,208 genes containing all CTG taxa was created. Alternative splits in the concatenated alignment were found using the NeighborNet method [82], and represented as a phylogenetic network with the SplitsTree software [83]. Using the software package Spectrum [84] we also performed a spectral analysis on this nucleotide alignment.

#### WGD clade

The WGD clade includes the genomes of *S. cerevisiae*, *S. paradoxus*, *S. mikatae*, *S. kudriavzevii*, *S. bayanus*, *S. castellii *and *C. glabrata. K. waltii *was selected as an outgroup. For a gene family to be retained, every gene within that family must locate every other family member (and nothing else) in a reciprocal BLASTP search (cutoff E-value of 10^-7^), be in single copy and contain a minimum of 4 taxa. We found 1,368 single gene families that met our criteria for supertree analysis. ML phylogenies were reconstructed for individual gene families as explained earlier. Phylogeny sets were also generated using Bayesian and distance based methods; [see additional file 4].

## Authors' contributions

DAF and GB were involved in the design phase. MEL & JES predicted genes in unannotated genomes. DAF & JES sourced putative orthologs. DAF performed all phylogenetic analyses. DAF and GB drafted the manuscript. All authors read and approved the final manuscript.

## Supplementary Material

Additional File 1 MSSA supertree derived from 4,805 fungal gene families. Bootstrap scores for all nodes are displayed. *Rhizopus oryzae *has been selected as an outgroup. The Basidiomycota and Ascomycota phyla form distinct clades. Subphyla and class clades are highlighted. Click here for file

Additional File 2 Descriptions of the 153 universally distributed genes. Click here for file

Additional File 3 Average consensus supertrees for WGD specific clade. For each of the 1,368 underlying gene families, fast evolving sites were categorised into 8 classes. Different site classes were systematically removed and phylogenies were reconstructed based on reduced alignments. (A) Fastest evolving sites (class 8) were removed. (B) The two fastest evolving site classes (classes 7 and 8) were removed. (C) The three fastest evolving site classes (classes 6, 7 and 8) were removed. Supertrees A and B group *S. castelli *and *C. glabrata *together, supertree C places *C. glabrata *at the base of the WGD clade. Click here for file

Additional File 4 Additional Methods and Results. Click here for file

Additional File 5 Bootstrap scores for phylogenetic Network. Click here for file

Additional File 6 Supertrees (AV (A), MRP (B) and MSSA (C)) derived from the strict gene family dataset that contains 809 genes. Bootstrap scores are shown at selected nodes. Overall there is agreement with supertrees derived from the larger (liberal) dataset. Click here for file
