# Supplementary material for: A fungal phylogeny based on 42 complete genomes derived from supertree and combined gene analysis
Source: BMC Evol Biol. 2006 Nov 22;6:99. doi: 10.1186/1471-2148-6-99 (PMC1679813; doi:10.1186/1471-2148-6-99)
Supplement: Additional File 2 — Descriptions of the 153 universally distributed genes. [file 1471-2148-6-99-S2.doc]

**additional file 2**: Descriptions of the 153 universally distributed genes used

in this analysis.

| Systematic Name | Standard Name | Function |
| --- | --- | --- |
| YJR065C | ARP3 | Essential component of the Arp2/3 complex |
| YER165W | PAB1 | Poly(A) binding protein |
| YLR438W | CAR2 | L-ornithine transaminase (OTAse) |
| YIL109C | SEC24 | Component of the Sec23p-Sec24p heterodimeric complex |
| YGR135W | PRE9 | 20S proteasome beta-type subunit |
| YER133W | GLC7 | Catalytic subunit of type 1 serine/threonine phosphatase |
| YER016W | BIM1 | Microtubule-binding protein |
| YPR176C | BET2 | Beta subunit of Type II geranylgeranyltransferase |
| YOR244W | ESA1 | Histone acetyltransferase catalytic subunit |
| YLR277C | YSH1 | Putative endonuclease |
| YIL043C | CBR1 | Microsomal cytochrome b reductase |
| YOL094C | RFC4 | Subunit of heteropentameric Replication factor C (RF-C) |
| YNL088W | TOP2 | Essential type II topoisomerase |
| YER125W | RSP5 | Ubiquitin-protein ligase |
| YER172C | BRR2 | RNA-dependent ATPase RNA helicase |
| YGL216W | KIP3 | Kinesin-related motor protein |
| YMR089C | YTA12 | Component |
| YGL194C | HOS2 | Histone deacetylase required for gene activation |
| YJL141C | YAK1 | Serine-threonine protein kinase |
| YHR102W | KIC1 | Protein kinase of the PAK/Ste20 kinase family |
| YNL064C | YDJ1 | Protein chaperone |
| YBR198C | TAF5 | Subunit (90 kDa) of TFIID and SAGA complexes |
| YBR229C | ROT2 | Glucosidase II catalytic subunit |
| YDR427W | RPN9 | Non-ATPase regulatory subunit of the 26S proteasome |
| YKL212W | SAC1 | Lipid phosphoinositide phosphatase of the ER and Golgi |
| YLR092W | SUL2 | High affinity sulfate permease |
| YDR226W | ADK1 | Adenylate kinase |
| YKR068C | BET3 | Hydrophilic protein that acts in conjunction with SNARE |
| YDR148C | KGD2 | Dihydrolipoyl transsuccinylase |
| YLL010C | PSR1 | Plasma membrane associated protein |
| YJR007W | SUI2 | Alpha subunit of the translation initiation factor eIF2 |
| YGR244C | LSC2 | Beta subunit of succinyl-CoA ligase |
| YOR326W | MYO2 | One of two type V myosins |
| YJL130C | URA2 | Bifunctional carbamoylphosphate synthetase |
| YER073W | ALD5 | Mitochondrial aldehyde dehydrogenase |
| YMR290C | HAS1 | ATP-dependent RNA helicase |
| YOL006C | TOP1 | Topoisomerase I |
| YLR086W | SMC4 | Subunit of the condensin complex |
| YOR184W | SER1 | 3-phosphoserine aminotransferase |
| YDR172W | SUP35 | Translation termination factor eRF3 |
| YJR045C | SSC1 | Mitochondrial matrix ATPase |
| YGR132C | PHB1 | Subunit of the prohibitin complex (Phb1p-Phb2p) |
| YDR021W | FAL1 | Nucleolar protein required for maturation of 18S rRNA |
| YJR068W | RFC2 | Subunit of heteropentameric Replication factor C (RF-C) |
| YDL164C | CDC9 | DNA ligase found in the nucleus and mitochondria |
| YBL076C | ILS1 | Cytoplasmic isoleucine-tRNA synthetase |
| YDR270W | CCC2 | Cu(+2)-transporting P-type ATPase |
| YDR212W | TCP1 | Alpha subunit of chaperonin-containing T-complex |
| YLL013C | PUF3 | Protein that regulates degradation of specific mRNAs |
| YGR218W | CRM1 | Major karyopherin |
| YPR066W | UBA3 | Protein that acts together with Ula1p to activate Rub1p |
| YMR216C | SKY1 | SR protein kinase (SRPK) |
| YDL102W | CDC2 | Catalytic subunit of DNA polymerase delta |
| YOR157C | PUP1 | Endopeptidase with trypsin-like activity |
| YMR167W | MLH1 | Protein required for mismatch repair in mitosis / meiosis |
| YKL145W | RPT1 | ATPases of the 19S regulatory particle of the 26S proteasome |
| YKL024C | URA6 | Uridylate kinase |
| YDL126C | CDC48 | ATPase in ER |
| YPR029C | APL4 | Gamma-adaptin |
| YPR119W | CLB2 | B-type cyclin involved in cell cycle progression |
| YER086W | ILV1 | Threonine deaminase |
| YKL001C | MET14 | Adenylylsulfate kinase |
| YHR008C | SOD2 | Manganese-containing superoxide dismutase |
| YHR031C | RRM3 | DNA helicase |
| YBL022C | PIM1 | Mitochondrial ATP-dependent protease |
| YDR120C | TRM1 | tRNA methyltransferase |
| YNL075W | IMP4 | Component of the SSU processome |
| YER023W | PRO3 | Delta 1-pyrroline-5-carboxylate reductase |
| YNL031C | HHT2 | One of two identical histone H3 proteins (see also HHT1) |
| YKL126W | YPK1 | Serine/threonine protein kinase |
| YDR107C | N/A | Uncharacterized ORF |
| YOR335C | ALA1 | Cytoplasmic alanyl-tRNA synthetase |
| YER013W | PRP22 | RNA-dependent ATPase/ATP-dependent RNA helicase |
| YER174C | GRX4 | glutathione-dependent oxidoreductase |
| YGR043C | N/A | Uncharacterized ORF |
| YDR170C | SEC7 | Guanine nucleotide exchange factor (GEF) |
| YPR141C | KAR3 | microtubule motor that functions in mitosis and meiosis |
| YCR093W | CDC39 | Component of the CCR4-NOT complex |
| YCR072C | RSA4 | WD-repeat protein involved in ribosome biogenesis |
| YBR127C | VMA2 | Subunit B subunit V1 peripheral membrane domain |
| YGL236C | MTO1 | Mitochondrial protein |
| YNL132W | KRE33 | Essential protein of unknown function |
| YIL094C | LYS12 | Homo-isocitrate dehydrogenase |
| YDR486C | VPS60 | Cytoplasmic and vacuolar membrane protein |
| YGR094W | VAS1 | Mitochondrial and cytoplasmic valyl-tRNA synthetase |
| YER015W | FAA2 | Long chain fatty acyl-CoA synthetase |
| YBL016W | FUS3 | Mitogen-activated protein kinase |
| YLR386W | VAC14 | Protein involved in regulated synthesis of PtdIns(3 |
| YBL064C | PRX1 | Mitochondrial peroxiredoxin (1-Cys Prx) |
| YER006W | NUG1 | GTPase that associates with nuclear 60S pre-ribosomes |
| YMR093W | UTP15 | Nucleolar protein |
| YFL038C | YPT1 | Ras-like small GTPase |
| YBR202W | CDC47 | Component of the hexameric MCM complex |
| YCL043C | PDI1 | Protein disulfide isomerase |
| YNR012W | URK1 | Uridine/cytidine kinase |
| YPL091W | GLR1 | Cytosolic and mitochondrial glutathione oxidoreductase |
| YPL063W | TIM50 | Constituent of the mitochondrial inner membrane presequence translocase (TIM23 complex) |
| YDL108W | KIN28 | Serine/threonine protein kinase |
| YLR447C | VMA6 | Subunit D of the five-subunit V0 integral membrane domain |
| YPL151C | PRP46 | Splicing factor |
| YMR297W | PRC1 | Vacuolar carboxypeptidase Y (proteinase C) |
| YNL209W | SSB2 | Cytoplasmic ATPase |
| YER148W | SPT15 | TATA-binding protein |
| YCR012W | PGK1 | 3-phosphoglycerate kinase |
| YFR031C | SMC2 | Component of the condensin complex |
| YPL217C | BMS1 | Essential conserved nucleolar GTP-binding protein |
| YPR080W | TEF1 | Translational elongation factor EF-1 alpha |
| YBR034C | HMT1 | Nuclear SAM-dependent mono- and asymmetric arginine dimethylating methyltransferase that modifies hnRNPs |
| YPR181C | SEC23 | GTPase-activating protein |
| YPL237W | SUI3 | Beta subunit of the translation initiation factor eIF2 |
| YDL145C | COP1 | Alpha subunit of COPI vesicle coatomer complex |
| YGL163C | RAD54 | DNA-dependent ATPase |
| YBL023C | MCM2 | Protein involved in DNA replication |
| YML035C | AMD1 | AMP deaminase |
| YDR238C | SEC26 | Essential beta-coat protein of the COPI coatomer |
| YOR317W | FAA1 | Long chain fatty acyl-CoA synthetase |
| YKL080W | VMA5 | Subunit of the eight-subunit V1 peripheral membrane domain |
| YOR270C | VPH1 | Subunit of vacuolar-ATPase V0 domain |
| YNL220W | ADE12 | Adenylosuccinate synthase |
| YNL161W | CBK1 | Serine/threonine protein kinase |
| YPR047W | MSF1 | Mitochondrial phenylalanyl-tRNA synthetase alpha subunit |
| YGR254W | ENO1 | Enolase I |
| YBR221C | PDB1 | E1 beta subunit of the pyruvate dehydrogenase complex |
| YDR099W | BMH2 | 14-3-3 protein |
| YBR084W | MIS1 | Mitochondrial C1-tetrahydrofolate synthase |
| YKR017C | N/A | Uncharacterized ORF |
| YPL028W | ERG10 | Acetyl-CoA C-acetyltransferase (acetoacetyl-CoA thiolase) |
| YDL137W | ARF2 | ADP-ribosylation factor |
| YGL091C | NBP35 | Essential nuclear protein |
| YBL050W | SEC17 | Peripheral membrane protein |
| YDR062W | LCB2 | Component of serine palmitoyltransferase |
| YCL059C | KRR1 | Essential nucleolar protein |
| YFL039C | ACT1 | Actin |
| YNL178W | RPS3 | Protein component of the small (40S) ribosomal subunit |
| YNL287W | SEC21 | Gamma subunit of coatomer |
| YLR244C | MAP1 | Methionine aminopeptidase |
| YAL038W | CDC19 | Pyruvate kinase |
| YGL022W | STT3 | Subunit of the oligosaccharyltransferase complex |
| YMR301C | ATM1 | Mitochondrial inner membrane transporter |
| YEL024W | RIP1 | Ubiquinol-cytochrome-c reductase |
| YCR057C | PWP2 | Conserved 90S pre-ribosomal component |
| YEL056W | HAT2 | Subunit of the Hat1p-Hat2p histone acetyltransferase complex |
| YIL074C | SER33 | 3-phosphoglycerate dehydrogenase |
| YJR064W | CCT5 | Subunit of the cytosolic chaperonin Cct ring complex |
| YGL062W | PYC1 | Pyruvate carboxylase isoform |
| YJL155C | FBP26 | Fructose-2 |
| YGR192C | TDH3 | Glyceraldehyde-3-phosphate dehydrogenase |
| YOR207C | RET1 | Second-largest subunit of RNA polymerase III |
| YJL050W | MTR4 | Dead-box family ATP dependent helicase |
| YLR069C | MEF1 | Mitochondrial elongation factor |
| YER107C | GLE2 | Component of the nuclear pore complex |
| YNL112W | DBP2 | Essential ATP-dependent RNA helicase |
| YLR195C | NMT1 | N-myristoyl transferase |
