# Supplementary material for: A fungal phylogeny based on 42 complete genomes derived from supertree and combined gene analysis
Source: BMC Evol Biol. 2006 Nov 22;6:99. doi: 10.1186/1471-2148-6-99 (PMC1679813; doi:10.1186/1471-2148-6-99)
Supplement: Additional File 4 — Additional Methods and Results. [file 1471-2148-6-99-S4.doc]

**Additional Methods and Results:**

**Additional Supertree Reconstruction Methods:**

In total 4,805 input trees were used are source data for the supertree analysis. In addition to the MRP and AV methods the most similar supertree analysis (MSSA) method [1] was also used. Using CLANN 3.0.3b1, 100 bootstrap resamplings were carried out on the input data.

Comparing the MRP (Figure 1A) and MSSA supertrees [see additional file 5] we observe some topological differences, including the inference that *A. nidulans* and *A. fumigatus* are sister taxa (79% BP), and *S. pombe* (the only member of the Schizosaccharomycetes) groups beside the Saccharomycotina (79% BP). Both MRP and AV supertrees (Figures 1 A&B) fail to make these inferences. Other differences include the inference that the {*K. waltii, S. kluyveri*} sub-clade is closer to the WGD clade than to the {*K. lactis, A. gossypii*} sub-clade [see additional file 5]. These inferences are identical to those made by the AV supertree. As described in the text we concluded that the AV method may be suffering from long branch attraction artifacts. The MSSA method does not consider branch lengths. These differences may be caused by input tree shape, although a previous study [2] did not reveal any obvious tree shape biases. The sister group relationships amongst the *Saccharomyces sensu stricto* species also differs between MSSA and MRP supertrees These incongruences highlight the pressing need for methods to reliably assess supertrees

.

**Strict database search to locate putative orthologous gene families**

To reduce the possibility that some of the input data used to construct supertrees contained paralogous sequences we utilised a second database search strategy (strict strategy). Our strict strategy insists that for a homologous gene family to be retained, every gene must locate every other family member (and nothing else) in a reciprocal BLASTP search (cutoff E-value of 10^-7^), be in single copy and contain a minimum of 4 taxa. After locating putative orthologous gene families (973 were located) post processing of families (alignments, PTP test etc) was performed in the same manner as described earlier to give a final dataset containing 809 gene families.

Using MultiPhyl [3] appropriate protein substitution models were selected and used to reconstruct maximum likelihood (ML) phylogenies for each individual gene family. Bootstrap resampling was carried out 100 times on each alignment and the results were summarised with the majority-rule consensus method with a threshold of 70%. These phylogenies were used as input data in our supertree analysis.

Supertree analysis was performed on this data and compared to supertrees generated from our larger dataset. Reassuringly there was a high degree of agreement between both datasets. In the MRP supertree [see additional file 6] there is a minor difference in the positioning of *C. lusitianiae* at the base of the CTG branch. The relative position of *S. nodorum* has also switched from the Eurotiomycetes to the Sordariomycetes. For the AV supertree the {*A. gossypii, K. lactis*} and {*K. waltii* *S. kluyveri*} clades appear to be more closely related to one another than to the WGD clade. *S. nodorum* now sits outside the Eurotiomycetes and and Sordariomycetes clades. Overall the MSSA supertree displays a similar topology when compared to the original MSSA supertree although branch supports are lower. The MSSA supertree groups *C. glabrata* and *S. castelli* as sister taxa.

**WGD clade**

Two additional phylogenetic reconstruction methods were used to infer sister group relationships amongst 1,368 protein families. Neighbor joining trees based on LogDet distances were generated using LDDist [4]. We also recoded the amino acid alignments into the six Dayhoff groups (C, STPAG, NDEQ, HRK, MILV and FWY). Recoded alignments were analyzed using the Bayesian criterion implemented in MrBayes V3.0b4 [5], using a 6X6 general time-reversible rate matrix. Among site variation in evolutionary rate was modeled with a proportion of sites allowed to vary. Four categories of rate variable sites were allowed, which were modeled by a discrete approximation to the gamma distribution. Each analysis used an MCMC chain that ran for 2 million generations sampled every 100th generation. The first 5,000 generations were discarded as the burn-in for each gene family. The resultant trees were summarised using the majority rule consensus with a 70% cutoff. Supertrees were reconstructed for all three phylogeny datasets and 100 bootstrap resamplings were performed on the data.

Both phylogeny sets were used to reconstruct an overall representative phylogeny with 3 supertree methods (AV, MRP and MSSA) as before. Irrespective of phylogeny set or supertree method used we always retrieved a strongly supported phylogeny with a topology identical to that displayed in Figure 3.

**Additional References**
