# Supplementary material for: A fungal phylogeny based on 42 complete genomes derived from supertree and combined gene analysis
Source: BMC Evol Biol. 2006 Nov 22;6:99. doi: 10.1186/1471-2148-6-99 (PMC1679813; doi:10.1186/1471-2148-6-99)
Supplement: Additional File 5 — Bootstrap scores for phylogenetic Network. [file 1471-2148-6-99-S5.doc]

TAXLABELS

[1] Aspergillus terreus

[2] Aspergillus oryzae

[3] Aspergillus fumigatus

[4] Aspergillus nidulans

[5] Coccidioides immitis

[6] Uncinocarpus reesii

[7] Histoplasma capsulatum

[8] Chaetomium globosum

[9] Podospora anserina

[10] Neurospora crassa

[11] Fusarium graminearum

[12] Fusarium verticillioides

[13] Trichoderma reesei

[14] Magnaporthe grisea

[15] Sclerotinia sclerotiorum

[16] Botrytis cinerea

[17] Stagonospora nodorum

[18] Coprinus cinereus

[19] Phanerochaete chrysosporium

[20] Ustilago maydis

[21] Cryptococcus neoformans

[22] Rhizopus oryzae

[23] Schizosaccharomyces pombe

[24] Yarrowia lipolytica

[25] Kluyveromyces waltii

[26] Saccharomyces kluyvermyces

[27] Ashbya gossypii

[28] Candida glabrata

[29] Saccharomyces castellii

[30] Saccharomyces cerevisiae

[31] Saccharomyces paradoxus

[32] Saccharomyces mikatae

[33] Saccharomyces bayanus

[34] Saccharomyces kudriavzevii

[35] Kluyveromyces lactis

[36] Candida albicans

[37] Candida dubliniensis

[38] Candida tropicalis

[39] Candida parapsilosis

[40] Candida lusitaniae

[41] Debaryomyces hansenii

[42] Candida guilliermondii

MATRIX

[1] 100.0 1 2 4 5 6 7 8 9 10 11 12 13 14 15 16 17 18 19 20 21 22 23 24 25 26 27 28 29 30 31 32 33 34 35 36 37 38 39 40 41 42,

[2] 100.0 1 2 5 6 7 8 9 10 11 12 13 14 15 16 17 18 19 20 21 22 23 24 25 26 27 28 29 30 31 32 33 34 35 36 37 38 39 40 41 42,

[3] 100.0 1 2 6,

[4] 100.0 1 2,

[5] 100.0 1,

[6] 100.0 1 2 3 5 6 7 8 9 10 11 12 13 14 15 16 17 18 19 20 21 22 23 24 25 26 27 28 29 30 31 32 33 34 35 36 37 38 39 40 41 42,

[7] 100.0 1 3,

[8] 100.0 1 2 3 4 5 6 7 8 9 10 11 12 13 14 15 16 18 19 20 21 22 23 24 25 26 27 28 29 30 31 32 33 34 35 36 37 38 39 40 41 42,

[9] 93.0 1 2 3 4 5 6 7 8 9 10 11 12 13 14 18 19 20 21 22 23 24 25 26 27 28 29 30 31 32 33 34 35 36 37 38 39 40 41 42,

[10] 100.0 1 2 3 4 5 6 7 8 9 10 11 14 18 19 20 21 22 23 24 25 26 27 28 29 30 31 32 33 34 35 36 37 38 39 40 41 42,

[11] 100.0 1 2 3 4 5 6 7,

[12] 100.0 1 2 3 4,

[13] 100.0 1 2 3 4 5 6 7 8 9 10 11 12 13 14 16 17 18 19 20 21 22 23 24 25 26 27 28 29 30 31 32 33 34 35 36 37 38 39 40 41 42,

[14] 100.0 1 2 3 4 5 6 7 8 9 10 11 12 13 14 17 18 19 20 21 22 23 24 25 26 27 28 29 30 31 32 33 34 35 36 37 38 39 40 41 42,

[15] 100.0 1 2 3 4 5 6 7 8 9 10 11 12 14 17 18 19 20 21 22 23 24 25 26 27 28 29 30 31 32 33 34 35 36 37 38 39 40 41 42,

[16] 91.0 1 2 3 4 5 6 7 14 17 18 19 20 21 22 23 24 25 26 27 28 29 30 31 32 33 34 35 36 37 38 39 40 41 42,

[17] 100.0 1 2 3 4 5 6 7 17 18 19 20 21 22 23 24 25 26 27 28 29 30 31 32 33 34 35 36 37 38 39 40 41 42,

[18] 100.0 1 2 3 4 5 6 7 8 9 10 11 12 13 14 15 17 18 19 20 21 22 23 24 25 26 27 28 29 30 31 32 33 34 35 36 37 38 39 40 41 42,

[19] 71.0 1 2 3 4 5 6 7 8 9 10 14 15 17 18 19 20 21 22 23 24 25 26 27 28 29 30 31 32 33 34 35 36 37 38 39 40 41 42,

[20] 88.0 1 2 3 4 5 6 7 15 17 18 19 20 21 22 23 24 25 26 27 28 29 30 31 32 33 34 35 36 37 38 39 40 41 42,

[21] 100.0 1 2 3 4 5 6 7 8 9 10 11 12 14 15 16 17 18 19 20 21 22 23 24 25 26 27 28 29 30 31 32 33 34 35 36 37 38 39 40 41 42,

[22] 100.0 1 2 3 4 5 6 7 8 9 10 14 15 16 17 18 19 20 21 22 23 24 25 26 27 28 29 30 31 32 33 34 35 36 37 38 39 40 41 42,

[23] 78.0 1 2 3 4 5 6 7 9 10 14 15 16 17 18 19 20 21 22 23 24 25 26 27 28 29 30 31 32 33 34 35 36 37 38 39 40 41 42,

[24] 100.0 1 2 3 4 5 6 7 15 16 17 18 19 20 21 22 23 24 25 26 27 28 29 30 31 32 33 34 35 36 37 38 39 40 41 42,

[25] 36.0 1 2 3 4 5 6 7 15 16 17 30 31 32 33 34,

[26] 75.0 1 2 3 4 5 6 15 16 17,

[27] 26.0 1 2 3 4 15 16 17,

[28] 100.0 1 2 3 4 5 6 7 8 9 10 11 13 14 15 16 17 18 19 20 21 22 23 24 25 26 27 28 29 30 31 32 33 34 35 36 37 38 39 40 41 42,

[29] 100.0 1 2 3 4 5 6 7 8 9 10 13 14 15 16 17 18 19 20 21 22 23 24 25 26 27 28 29 30 31 32 33 34 35 36 37 38 39 40 41 42,

[30] 69.0 1 2 3 4 5 6 7 9 10 13 14 15 16 17 18 19 20 21 22 23 24 25 26 27 28 29 30 31 32 33 34 35 36 37 38 39 40 41 42,

[31] 45.0 1 2 3 4 5 6 7 13 15 16 17 18 19 20 21 22 23 24 25 26 27 28 29 30 31 32 33 34 35 36 37 38 39 40 41 42,

[32] 100.0 1 2 3 4 5 6 7 8 9 10 12 13 14 15 16 17 18 19 20 21 22 23 24 25 26 27 28 29 30 31 32 33 34 35 36 37 38 39 40 41 42,

[33] 100.0 1 2 3 4 5 6 7 12 13 15 16 17 18 19 20 21 22 23 24 25 26 27 28 29 30 31 32 33 34 35 36 37 38 39 40 41 42,

[34] 52.0 1 2 3 4 5 6 7 12 13 15 16 17 25 26 27 28 29 30 31 32 33 34 35 36 37 38 39 40 41 42,

[35] 30.0 1 2 3 4 5 6 7 12 13 15 16 17 30 32 33 34,

[36] 75.0 1 2 3 4 6 12 13 15 16 17,

[37] 100.0 1 2 3 4 5 6 7 9 10 11 12 13 14 15 16 17 18 19 20 21 22 23 24 25 26 27 28 29 30 31 32 33 34 35 36 37 38 39 40 41 42,

[38] 99.0 1 2 3 4 5 6 7 10 11 12 13 14 15 16 17 18 19 20 21 22 23 24 25 26 27 28 29 30 31 32 33 34 35 36 37 38 39 40 41 42,

[39] 100.0 1 2 3 4 5 6 7 11 12 13 14 15 16 17 18 19 20 21 22 23 24 25 26 27 28 29 30 31 32 33 34 35 36 37 38 39 40 41 42,

[40] 100.0 1 2 3 4 5 6 7 8 10 11 12 13 14 15 16 17 18 19 20 21 22 23 24 25 26 27 28 29 30 31 32 33 34 35 36 37 38 39 40 41 42,

[41] 78.0 1 2 3 4 5 6 7 8 11 12 13 15 16 17 18 19 20 21 22 23 24 25 26 27 28 29 30 31 32 33 34 35 36 37 38 39 40 41 42,

[42] 100.0 1 2 3 4 5 6 7 8 9 11 12 13 14 15 16 17 18 19 20 21 22 23 24 25 26 27 28 29 30 31 32 33 34 35 36 37 38 39 40 41 42,

[43] 92.0 1 2 3 4 5 6 7 8 9 11 12 13 15 16 17 18 19 20 21 22 23 24 25 26 27 28 29 30 31 32 33 34 35 36 37 38 39 40 41 42,

[44] 100.0 1 2 3 4 5 6 7 8 9 10 11 12 13 15 16 17 18 19 20 21 22 23 24 25 26 27 28 29 30 31 32 33 34 35 36 37 38 39 40 41 42,

[45] 22.0 1 2 3 4 5 6 7 8 9 10 11 12 13 15 16 17 18 19 20 21 23 24 25 26 27 28 29 30 31 32 33 34 35 36 37 38 39 40 41 42,

[46] 12.0 1 2 3 4 5 6 7 8 9 10 11 12 13 15 16 17 20 23 24 25 26 27 28 29 30 31 32 33 34 35 36 37 38 39 40 41 42,

[47] 100.0 1 2 3 4 5 6 7 8 9 10 11 12 13 14 15 16 17 18 19 20 21 23 24 25 26 27 28 29 30 31 32 33 34 35 36 37 38 39 40 41 42,

[48] 66.0 1 2 3 4 5 6 7 8 9 10 11 12 13 14 15 16 17 20 23 24 25 26 27 28 29 30 31 32 33 34 35 36 37 38 39 40 41 42,

[49] 100.0 1 2 3 4 5 6 7 8 9 10 11 12 13 14 15 16 17 23 24 25 26 27 28 29 30 31 32 33 34 35 36 37 38 39 40 41 42,

[50] 100.0 1 2 3 4 5 6 7 8 9 10 11 12 13 14 15 16 17 24 25 26 27 28 29 30 31 32 33 34 35 36 37 38 39 40 41 42,

[51] 33.0 1 2 3 4 5 6 7 8 9 10 11 12 13 14 15 16 17 32 33 34,

[52] 100.0 1 2 3 4 5 6 7 8 9 10 11 12 13 14 15 16 17,

[53] 45.0 1 2 3 4 5 6 8 9 10 11 12 13 14 15 16 17,

[54] 100.0 1 2 3 4 5 6 7 8 9 10 11 12 13 14 15 16 17 18 19 20 22 23 24 25 26 27 28 29 30 31 32 33 34 35 36 37 38 39 40 41 42,

[55] 100.0 1 2 3 4 5 6 7 8 9 10 11 12 13 14 15 16 17 18 20 22 23 24 25 26 27 28 29 30 31 32 33 34 35 36 37 38 39 40 41 42,

[56] 100.0 1 2 3 4 5 6 7 8 9 10 11 12 13 14 15 16 17 20 22 23 24 25 26 27 28 29 30 31 32 33 34 35 36 37 38 39 40 41 42,

[57] 100.0 1 2 3 4 5 6 7 8 9 10 11 12 13 14 15 16 17 22 23 24 25 26 27 28 29 30 31 32 33 34 35 36 37 38 39 40 41 42,

[58] 86.0 1 2 3 4 5 6 7 8 9 10 11 12 13 14 15 16 17 22 24 25 26 27 28 29 30 31 32 33 34 35 36 37 38 39 40 41 42,

[59] 32.0 1 2 3 4 5 6 7 8 9 10 11 12 13 14 15 16 17 22 25 26 27 28 29 30 31 32 33 34 35 36 37 38 39 40 41 42,

[60] 100.0 1 2 3 4 5 6 7 8 9 10 11 12 13 14 15 16 17 18 20 21 22 23 24 25 26 27 28 29 30 31 32 33 34 35 36 37 38 39 40 41 42,

[61] 100.0 1 2 3 4 5 6 7 8 9 10 11 12 13 14 15 16 17 20 21 22 23 24 25 26 27 28 29 30 31 32 33 34 35 36 37 38 39 40 41 42,

[62] 99.0 1 2 3 4 5 6 7 8 9 10 11 12 13 14 15 16 17 21 22 23 24 25 26 27 28 29 30 31 32 33 34 35 36 37 38 39 40 41 42,

[63] 17.0 1 2 3 4 5 6 7 8 9 10 11 12 13 14 15 16 17 21 22 24 25 26 27 28 29 30 31 32 33 34 35 36 37 38 39 40 41 42,

[64] 100.0 1 2 3 4 5 6 7 8 9 10 11 12 13 14 15 16 17 19 20 21 22 23 24 25 26 27 28 29 30 31 32 33 34 35 36 37 38 39 40 41 42,

[65] 76.0 1 2 3 4 5 6 7 8 9 10 11 12 13 14 15 16 17 19 21 22 23 24 25 26 27 28 29 30 31 32 33 34 35 36 37 38 39 40 41 42,

[66] 100.0 1 2 3 4 5 6 7 8 9 10 11 12 13 14 15 16 17 18 19 21 22 23 24 25 26 27 28 29 30 31 32 33 34 35 36 37 38 39 40 41 42,

[67] 31.0 1 2 3 4 5 6 7 8 9 10 11 12 13 14 15 16 17 18 19 21 22 24 25 26 27 28 29 30 31 32 33 34 35 36 37 38 39 40 41 42,

[68] 100.0 1 2 3 4 5 6 7 8 9 10 11 12 13 14 15 16 17 18 19 20 21 22 24 25 26 27 28 29 30 31 32 33 34 35 36 37 38 39 40 41 42,

[69] 97.0 1 2 3 4 5 6 7 8 9 10 11 12 13 14 15 16 17 18 19 20 21 22 25 26 27 28 29 30 31 32 33 34 35 36 37 38 39 40 41 42,

[70] 67.0 1 2 3 4 5 6 7 8 9 10 11 12 13 14 15 16 17 18 19 20 21 22,

[71] 24.0 1 2 3 4 8 9 10 11 12 13 14 15 16 17 18 19 20 21 22,

[72] 100.0 1 2 3 4 5 6 7 8 9 10 11 12 13 14 15 16 17 18 19 20 21 22 23 25 26 27 28 29 30 31 32 33 34 35 36 37 38 39 40 41 42,

[73] 74.0 1 2 3 4 5 6 7 8 9 10 11 12 13 14 15 16 17 18 19 20 21 22 23 25 26 27 28 29 30 31 32 33 34 35,

[74] 30.0 1 2 3 4 5 6 7 8 9 10 11 12 13 14 15 16 17 18 19 20 21 22 23 34,

[75] 100.0 1 2 3 4 5 6 7 8 9 10 11 12 13 14 15 16 17 18 19 20 21 22 23,

[76] 100.0 1 2 3 4 5 6 7 8 9 10 11 12 13 14 15 16 17 18 19 20 21 22 23 24 25 26 27 28 29 30 31 32 33 34 35 36 37 38 40 41 42,

[77] 100.0 1 2 3 4 5 6 7 8 9 10 11 12 13 14 15 16 17 18 19 20 21 22 23 24 25 26 27 28 29 30 31 32 33 34 35 36 38 40 41 42,

[78] 100.0 1 2 3 4 5 6 7 8 9 10 11 12 13 14 15 16 17 18 19 20 21 22 23 24 25 26 27 28 29 30 31 32 33 34 35 38 40 41 42,

[79] 100.0 1 2 3 4 5 6 7 8 9 10 11 12 13 14 15 16 17 18 19 20 21 22 23 24 25 26 27 28 29 30 31 32 33 34 35 40 41 42,

[80] 99.0 1 2 3 4 5 6 7 8 9 10 11 12 13 14 15 16 17 18 19 20 21 22 23 24 25 26 27 28 29 30 31 32 33 34 35 40,

[81] 100.0 1 2 3 4 5 6 7 8 9 10 11 12 13 14 15 16 17 18 19 20 21 22 23 24 25 26 27 28 29 30 31 32 33 34 35,

[82] 100.0 1 2 3 4 5 6 7 8 9 10 11 12 13 14 15 16 17 18 19 20 21 22 23 24,

[83] 100.0 1 2 3 4 5 6 7 8 9 10 11 12 13 14 15 16 17 18 19 20 21 22 23 24 25 26 27 28 29 30 31 32 33 34 35 36 38 39 40 41 42,

[84] 100.0 1 2 3 4 5 6 7 8 9 10 11 12 13 14 15 16 17 18 19 20 21 22 23 24 25 26 27 28 29 30 31 32 33 34 35 38 39 40 41 42,

[85] 100.0 1 2 3 4 5 6 7 8 9 10 11 12 13 14 15 16 17 18 19 20 21 22 23 24 25 26 27 28 29 30 31 32 33 34 35 39 40 41 42,

[86] 100.0 1 2 3 4 5 6 7 8 9 10 11 12 13 14 15 16 17 18 19 20 21 22 23 24 25 26 27 28 29 30 31 32 33 34 35 39,

[87] 64.0 1 2 3 4 5 6 7 8 9 10 11 12 13 14 15 16 17 18 19 20 21 22 23 24 39,

[88] 100.0 1 2 3 4 5 6 7 8 9 10 11 12 13 14 15 16 17 18 19 20 21 22 23 24 25 26 27 28 29 30 31 32 33 34 35 37 38 39 40 41 42,

[89] 99.0 1 2 3 4 5 6 7 8 9 10 11 12 13 14 15 16 17 18 19 20 21 22 23 24 25 26 27 28 29 30 31 32 33 34 35 37 39 40 41 42,

[90] 100.0 1 2 3 4 5 6 7 8 9 10 11 12 13 14 15 16 17 18 19 20 21 22 23 24 25 26 27 28 29 30 31 32 33 34 35 37 39,

[91] 81.0 1 2 3 4 5 6 7 8 9 10 11 12 13 14 15 16 17 18 19 20 21 22 23 24 30 32 33 34 37 39,

[92] 100.0 1 2 3 4 5 6 7 8 9 10 11 12 13 14 15 16 17 18 19 20 21 22 23 24 25 26 27 28 29 30 31 32 33 34 35 36 37 39 40 41 42,

[93] 88.0 1 2 3 4 5 6 7 8 9 10 11 12 13 14 15 16 17 18 19 20 21 22 23 24 25 26 27 28 29 30 31 32 33 34 35 36 37 39,

[94] 33.0 1 2 3 4 5 6 7 8 9 10 11 12 13 14 15 16 17 18 19 20 21 22 23 24 25 28 29 30 31 32 33 34 35 36 37 39,

[95] 100.0 1 2 3 4 5 6 7 8 9 10 11 12 13 14 15 16 17 18 19 20 21 22 23 24 25 26 27 28 29 30 31 32 33 34 35 36 37 38 39 40 41,

[96] 100.0 1 2 3 4 5 6 7 8 9 10 11 12 13 14 15 16 17 18 19 20 21 22 23 24 25 26 27 28 29 30 31 32 33 34 35 36 37 38 39 40,

[97] 67.0 1 2 3 4 5 6 7 8 9 10 11 12 13 14 15 16 17 18 19 20 21 22 23 24 25 26 27 28 29 30 31 32 33 34 35 36 37 38 39,

[98] 83.0 1 2 3 4 5 6 7 8 9 10 11 12 13 14 15 16 17 18 19 20 21 22 23 24 28 29 30 31 32 33 34 35 36 37 38 39,

[99] 100.0 1 2 3 4 5 6 7 8 9 10 11 12 13 14 15 16 17 18 19 20 21 22 23 24 25 26 27 28 29 30 31 32 33 34 35 36 37 38 39 40 42,

[100] 100.0 1 2 3 4 5 6 7 8 9 10 11 12 13 14 15 16 17 18 19 20 21 22 23 24 25 26 27 28 29 30 31 32 33 34 35 36 37 38 39 42,

[101] 100.0 1 2 3 4 5 6 7 8 9 10 11 12 13 14 15 16 17 18 19 20 21 22 23 24 25 26 27 28 29 30 31 32 33 34 35 36 37 38 39 41 42,

[102] 32.0 1 2 3 4 5 6 7 8 9 10 11 12 13 14 15 16 17 18 19 20 21 22 23 24 25 26 28 29 30 31 32 33 34 35 36 37 38 39 41 42,

[103] 100.0 1 2 3 4 5 6 7 8 9 10 11 12 13 14 15 16 17 18 19 20 21 22 23 24 25 26 28 29 30 31 32 33 34 35 36 37 38 39 40 41 42,

[104] 100.0 1 2 3 4 5 6 7 8 9 10 11 12 13 14 15 16 17 18 19 20 21 22 23 24 28 29 30 31 32 33 34 36 37 38 39 40 41 42,

[105] 73.0 1 2 3 4 5 6 7 8 9 10 11 12 13 14 15 16 17 18 19 20 21 22 23 24 30 32 33 34 36 37 38 39 40 41 42,

[106] 84.0 1 2 3 4 5 6 7 8 9 10 11 12 13 14 15 16 17 18 19 20 21 22 23 24 32 33 34 36 37 38 39 40 41 42,

[107] 100.0 1 2 3 4 5 6 7 8 9 10 11 12 13 14 15 16 17 18 19 20 21 22 23 24 36 37 38 39 40 41 42,

[108] 100.0 1 2 3 4 5 6 7 8 9 10 11 12 13 14 15 16 17 18 19 20 21 22 23 24 25 27 28 29 30 31 32 33 34 35 36 37 38 39 40 41 42,

[109] 99.0 1 2 3 4 5 6 7 8 9 10 11 12 13 14 15 16 17 18 19 20 21 22 23 24 27 28 29 30 31 32 33 34 35 36 37 38 39 40 41 42,

[110] 20.0 1 2 3 4 5 6 7 8 9 10 11 12 13 14 15 16 17 18 19 20 21 22 23 24 27 32 33 34 36 37 38 39 40 41 42,

[111] 100.0 1 2 3 4 5 6 7 8 9 10 11 12 13 14 15 16 17 18 19 20 21 22 23 24 27 34 36 37 38 39 40 41 42,

[112] 85.0 1 2 3 4 5 6 7 8 9 10 11 12 13 14 15 16 17 18 19 20 21 22 23 24 27 36 37 38 39 40 41 42,

[113] 100.0 1 2 3 4 5 6 7 8 9 10 11 12 13 14 15 16 17 18 19 20 21 22 23 24 26 27 28 29 30 31 32 33 34 35 36 37 38 39 40 41 42,

[114] 100.0 1 2 3 4 5 6 7 8 9 10 11 12 13 14 15 16 17 18 19 20 21 22 23 24 25 26 27 28 29 30 31 32 33 34 36 37 38 39 40 41 42,

[115] 100.0 1 2 3 4 5 6 7 8 9 10 11 12 13 14 15 16 17 18 19 20 21 22 23 24 25 26 27 29 30 31 32 33 34 35 36 37 38 39 40 41 42,

[116] 45.0 1 2 3 4 5 6 7 8 9 10 11 12 13 14 15 16 17 18 19 20 21 22 23 24 25 26 27 32 33 34 35 36 37 38 39 40 41 42,

[117] 74.0 1 2 3 4 5 6 7 8 9 10 11 12 13 14 15 16 17 18 19 20 21 22 23 24 25 26 27 33 34 35 36 37 38 39 40 41 42,

[118] 94.0 1 2 3 4 5 6 7 8 9 10 11 12 13 14 15 16 17 18 19 20 21 22 23 24 25 26 27 34 35 36 37 38 39 40 41 42,

[119] 100.0 1 2 3 4 5 6 7 8 9 10 11 12 13 14 15 16 17 18 19 20 21 22 23 24 25 26 27 35 36 37 38 39 40 41 42,

[120] 100.0 1 2 3 4 5 6 7 8 9 10 11 12 13 14 15 16 17 18 19 20 21 22 23 24 25 26 27 28 30 31 32 33 34 35 36 37 38 39 40 41 42,

[121] 58.0 1 2 3 4 5 6 7 8 9 10 11 12 13 14 15 16 17 18 19 20 21 22 23 24 25 26 27 28 33 34 35 36 37 38 39 40 41 42,

[122] 100.0 1 2 3 4 5 6 7 8 9 10 11 12 13 14 15 16 17 18 19 20 21 22 23 24 25 26 27 28 35 36 37 38 39 40 41 42,

[123] 100.0 1 2 3 4 5 6 7 8 9 10 11 12 13 14 15 16 17 18 19 20 21 22 23 24 25 26 27 28 29 30 32 33 34 35 36 37 38 39 40 41 42,

[124] 100.0 1 2 3 4 5 6 7 8 9 10 11 12 13 14 15 16 17 18 19 20 21 22 23 24 25 26 27 28 29 32 33 34 35 36 37 38 39 40 41 42,

[125] 99.0 1 2 3 4 5 6 7 8 9 10 11 12 13 14 15 16 17 18 19 20 21 22 23 24 25 26 27 28 29 33 34 35 36 37 38 39 40 41 42,

[126] 82.0 1 2 3 4 5 6 7 8 9 10 11 12 13 14 15 16 17 18 19 20 21 22 23 24 25 26 27 28 29 34 35 36 37 38 39 40 41 42,

[127] 100.0 1 2 3 4 5 6 7 8 9 10 11 12 13 14 15 16 17 18 19 20 21 22 23 24 25 26 27 28 29 35 36 37 38 39 40 41 42,

[128] 100.0 1 2 3 4 5 6 7 8 9 10 11 12 13 14 15 16 17 18 19 20 21 22 23 24 25 26 27 28 29 31 32 33 34 35 36 37 38 39 40 41 42,

[129] 81.0 1 2 3 4 5 6 7 8 9 10 11 12 13 14 15 16 17 18 19 20 21 22 23 24 25 26 27 28 29 31 35 36 37 38 39 40 41 42,

[130] 100.0 1 2 3 4 5 6 7 8 9 10 11 12 13 14 15 16 17 18 19 20 21 22 23 24 25 26 27 28 29 30 31 33 34 35 36 37 38 39 40 41 42,

[131] 100.0 1 2 3 4 5 6 7 8 9 10 11 12 13 14 15 16 17 18 19 20 21 22 23 24 25 26 27 28 29 30 31 32 34 35 36 37 38 39 40 41 42,

[132] 100.0 1 2 3 4 5 6 7 8 9 10 11 12 13 14 15 16 17 18 19 20 21 22 23 24 25 26 27 28 29 30 31 32 33 35 36 37 38 39 40 41 42,

[133] 100.0 1 2 3 4 5 6 8 9 10 11 12 13 14 15 16 17 18 19 20 21 22 23 24 25 26 27 28 29 30 31 32 33 34 35 36 37 38 39 40 41 42,

[134] 100.0 1 2 3 4 6 8 9 10 11 12 13 14 15 16 17 18 19 20 21 22 23 24 25 26 27 28 29 30 31 32 33 34 35 36 37 38 39 40 41 42,

[135] 64.0 1 2 3 4 8 9 10 11 12 13 14 15 16 17 18 19 20 21 22 23 24 25 26 27 28 29 30 31 32 33 34 35 36 37 38 39 40 41 42,

[136] 100.0 1 2 3 4 6 7 8 9 10 11 12 13 14 15 16 17 18 19 20 21 22 23 24 25 26 27 28 29 30 31 32 33 34 35 36 37 38 39 40 41 42,

[137] 100.0 1 2 3 4 7 8 9 10 11 12 13 14 15 16 17 18 19 20 21 22 23 24 25 26 27 28 29 30 31 32 33 34 35 36 37 38 39 40 41 42,

[138] 100.0 1 2 3 4 5 7 8 9 10 11 12 13 14 15 16 17 18 19 20 21 22 23 24 25 26 27 28 29 30 31 32 33 34 35 36 37 38 39 40 41 42,

[139] 100.0 1 3 4 5 6 7 8 9 10 11 12 13 14 15 16 17 18 19 20 21 22 23 24 25 26 27 28 29 30 31 32 33 34 35 36 37 38 39 40 41 42,
